# Evaluation Framework for Bruise Detection: Systematic ALS/White-Light Training and Skin-Tone Balancing with Deep Learning

**DOI:** 10.3390/s26103215

**Published:** 2026-05-19

**Authors:** Kiyarash Aminfar, Katherine Scafide, Janusz Wojtusiak, David Lattanzi

**Affiliations:** 1 Sid and Reva Dewberry Department of Civil, Environmental, and Infrastructure Engineering, College of Engineering and Computing, George Mason University, Fairfax, VA 22030, USA; 2 School of Nursing, College of Public Health, George Mason University, Fairfax, VA 22030, USA; 3 Department of Global and Community Health, College of Public Health, George Mason University, Fairfax, VA 22030, USA

**Keywords:** transfer learning, computer vision, medical image analysis, injury detection

## Abstract

Accurate and consistent forensic bruise assessment is critical in ensuring positive clinical and legal outcomes for victims of violence. In this study, a framework for automated bruise detection is presented that, for the first time, integrates narrowband alternate-light-source (ALS) forensic imaging and ambient white light imaging. This evaluation framework is designed to address long-standing issues with respect to equitable performance across skin tones and lighting scenarios via a combination of novel model diagnostic strategies. In particular, skin-tone balancing during training and testing, threshold-sensitivity analysis, and embedding-similarity partitioning are employed to quantify the model robustness and deployment trade-offs that arise in forensic image analysis. Models were implemented with ImageNet-pretrained backbones and trained on a unique, multi-annotator full-consensus dataset comprising both white-light and ALS (415 nm and 450 nm) images. The protocol emphasizes three axes of operational relevance: (1) illumination composition in training (W/ALS ratio); (2) subgroup fairness via targeted balancing; and (3) model operating-point selection (confidence and IoU thresholds) informed by confidence-stability metrics and bootstrapped uncertainty estimates. Systematic W/ALS ratio sweeps indicate peak accuracy under ALS-dominant training and declining performance as the proportion of white-light images increases within the training set. Skin-tone balancing reduced failure rates for darker skin tones but increased overprediction in some demographic subgroups. Embedding-similarity and seen/unseen injury analyses demonstrate inflated generalization under image-level partitioning. Ultimately, the findings suggest that future researchers and developers should employ injury-level data partitioning and ensure a weighted balance of ALS images during training.

## 1. Introduction

Bruises form as a result of mechanical trauma that damages the skin and underlying tissues, leading to vascular rupture and localized bleeding. In forensic investigations, bruise patterns can serve as indicators of physical trauma, facilitating the distinction between accidental and non-accidental injuries. For example, the size, shape, and distribution of bruises are often used to identify potential signs of elder abuse [1]. Accurate bruise detection and analysis are essential in both clinical and forensic contexts, where estimating bruise age and other characteristics can aid in establishing timelines and potential causes of injury.

Forensic photography is widely employed for documenting bruises, with studies exploring how imaging modalities influence bruise visibility and characterization. Bruise size and intensity are commonly used to infer the extent of vascular damage, with larger bruises often suggesting more severe trauma [2]. However, these parameters are unreliable indicators of applied force due to inter-individual variability in skin thickness, clotting mechanisms, and healing responses. Visual assessment is further complicated by significant differences in bruise formation and progression across individuals, limiting the accuracy of injury severity and age estimations [3,4,5].

### 1.1. Emerging Technologies in Bruise Detection

Due to the limitations of subjective visual assessments, forensic and medical research have increasingly investigated advanced imaging modalities and computational techniques for bruise detection and analysis. Alternate Light Source (ALS) technology enhances forensic injury documentation by using wavelength-specific illumination to improve bruise visibility [6,7,8]. ALS devices employ selective wavelength light emission combined with optical filters chosen based on the absorption spectra of hemoglobin and its breakdown products. The increased contrast between bruised and non-bruised tissue facilitates the detection of subcutaneous injuries that may not be visible under standard lighting [9]. ALS-based visibility scales have demonstrated high interrater reliability and correlate well with bruise size and contrast [10]. However, wavelengths exceeding 515 nm reduce detection accuracy [11]. Studies using pigskin models and histological confirmation found that ALS-enhanced imaging may also highlight non-bruised regions, leading to potential false positives and raising concerns about specificity [8], thereby emphasizing the need for refined forensic protocols [12].

Given these challenges, computer-aided measurement techniques have been developed to improve bruise quantification in forensic applications [4]. A study utilizing ImageJ-based automated image processing demonstrated that RGB2 yielded more reliable and objective bruise size measurements, outperforming manual assessments [13]. While this approach may enhance forensic accuracy, it lacks adaptability for real-time bruise detection, highlighting the necessity for machine learning-driven object detection models.

While traditional image processing techniques improve bruise quantification, their reliance on predefined parameters limits adaptability to variations in lighting conditions, skin pigmentation, and bruise progression. Deep Learning (DL) techniques are transforming forensic and medical imaging by enabling automated injury pattern analysis [14]. Object detection models have demonstrated high diagnostic accuracy across various medical applications, including skin cancer detection and brain lesion segmentation, improving both diagnostic precision and clinical decision-making [15]. These advancements are exemplified by the major role Convolutional Neural Network (CNN) plays in image classification and object detection across various forensic science applications [16], with further evidence from medical imaging indicating that DL models achieve diagnostic performance comparable to healthcare professionals in image-based disease detection [17].

Despite these advancements, dataset limitations remain a critical challenge in forensic bruise analysis. Many existing DL models are trained predominantly on image datasets derived from European and East Asian populations, resulting in potential diagnostic biases due to underrepresentation of darker skin tones [18]. Ensuring equitable representation in training datasets is essential to ensure the practical applicability of DL-based forensic tools. Additionally, the scarcity of large, annotated datasets specific to bruise detection limits the ability of AI models to generalize across diverse demographic and physiological characteristics.

### 1.2. Object Detection Models in Bruise Analysis

Advancements in object detection have led to the development of models optimized for speed, accuracy, and computational efficiency. Widely adopted architectures in forensic and medical image analysis include Faster R-CNN, FCOS, RetinaNet, YOLO, and EfficientDet [15]. Faster R-CNN integrates a Region Proposal Network (RPN) to balance detection speed and accuracy, making it suitable for real-time forensic applications. In crime scene analysis using high-performance GPUs, it has demonstrated 74.33% accuracy with a mean detection time of 0.12 s per image [19].

Other architectures address specific detection challenges. RetinaNet employs a focal loss function to mitigate class imbalance, whereas FCOS simplifies the model by eliminating the need for anchor boxes. YOLO (You Only Look Once) is advantageous for real-time applications due to its high inference speed. EfficientDet provides a scalable and computationally efficient framework that optimizes performance across different resource constraints. Further details on the selected object detection models are provided in Section 3.2.

### 1.3. Study Goal and Contributions

Forensic applications require not only accurate models but reproducible, operational validation that quantifies how acquisition, dataset composition, and decision thresholds impact real-world performance. Critical deployment risks that are frequently overlooked include (1) illumination/filter-dependent performance shifts when models are trained on mixtures of white light (W) and alternate light source (ALS) images; (2) subgroup performance differentials arising from uneven skin-tone representation and data balancing strategies; and (3) optimistic generalization estimates resulting from image-level partitioning when multiple images of the same injury appear across splits. These gaps constrain the practical utility for forensic practitioners and hinder methodologically comparable evaluations across studies.

To address these gaps, an operational evaluation and validation protocol is developed and applied to a curated, multi-annotator full-consensus dataset containing images captured under varying lighting conditions and optical filtration, including ALS configurations and standard white light. Model performance is assessed using established medical-imaging metrics, such as accuracy, precision, recall, mean average precision (mAP), false/true positive rates (FP/TP), intersection over union (IoU), and inference time [15]. These metrics provide empirical insight into the effectiveness of different model architectures for bruise detection while exposing prediction biases.

The study provides four interlocking contributions:

(1) Dual-modality detection evaluation: A bruise detection and documentation model capable of handling both ambient white light and specialized narrow-band ALS forensic images with comparable levels of performance.
(2) Illumination-composition diagnostics: Systematic W/ALS training-ratio sweeps quantify the influence of ALS exposure during training on detection sensitivity, precision, and localization stability. The analysis identifies regimes in which ALS-dominant training materially improves AP and regimes in which white-light inclusion degrades performance, thereby informing acquisition priorities for deployment.
(3) Subgroup fairness and localization trade-off analysis: Fairness for detection tasks is operationalized by measuring failure-rate reduction, localization variance, and overprediction frequency across skin-tone strata under targeted balancing strategies. This approach exposes trade-offs between improved sensitivity for darker skin tones and increased spatial uncertainty, informing dataset curation and annotation guidelines.
(4) Partitioning diagnostics via embedding-similarity and seen/unseen evaluation: Backbone-derived image embeddings and Euclidean-similarity quantiles are used to quantify the extent to which image-level partitioning inflates apparent generalization. Stratified analyses (seen → unseen and top → bottom similarity) with bootstrapped confidence intervals demonstrate substantive mAP declines on less-similar or unseen injuries.

Methodologically, these contributions are implemented within a reproducible pipeline: ImageNet-pretrained backbones with multi-annotator full-consensus labels, identical preprocessing and augmentation across models, and explicit threshold-sensitivity sweeps (confidence and IoU) with bootstrap uncertainty estimates. The protocol is applied to three canonical object-detection architectures (Faster R-CNN, RetinaNet, FCOS). Quantitative comparisons are accompanied by a structured failure-mode taxonomy (Perfect, Underpredict, Overpredict, Failed) to characterize error modes.

Rather than identifying a single superior detector, the primary outcome is an evaluation and operationalization workflow that (a) illustrates dataset- and model-dependent trade-offs, (b) prescribes validation best practices (including injury-level or embedding-stratified testing), and (c) yields deployment-oriented recommendations (e.g., ALS prioritization and model-specific thresholding).

## 2. Related Works

Automated bruise detection, unlike established forensic and medical injury documentation methods (e.g., forensic photography, alternate light sources), remains largely unexplored due to inherent challenges. This section reviews existing AI-driven bruise detection research and methodologically similar domains, such as feature extraction, segmentation, and classification. Given the scarcity of dedicated forensic bruise detection studies, insights from medical imaging, agricultural bruise detection, and structural defect analysis are explored to inform the development of more robust and generalizable forensic AI models.

Despite advancements in AI for medical imaging, automated bruise detection research is limited. One study explored CNN-based bruise age classification using Inception V3, ResNet-50, and MobileNet architectures and reported 97% accuracy, far exceeding the typical accuracy observed in forensic assessments [20]. While this highlights DL’s feasibility for bruise analysis, further validation and adaptation are crucial to address variations in imaging modalities, lighting conditions, and inter-individual differences in bruise appearance.

### 2.1. AI in Medical Imaging

AI has been widely applied in dermatology, particularly for the automated diagnosis and differentiation of skin malignancies [21,22]. Leveraging large datasets, DL models achieve high accuracy in distinguishing benign and malignant lesions [17], with transfer learning enabling effective skin lesion classification even on smaller datasets [23]. Systematic reviews of AI in dermatology between 2011–2021 [24] and 2020–2022 [25] highlight a focus on detection, segmentation, and classification tasks.

Techniques developed for non-traumatic dermatological lesions, such as feature extraction, transfer learning, and model fine-tuning, may be transferable to forensic bruise analysis. However, forensic applications require modifications to account for variations in lighting, skin pigmentation, and bruise appearance over time. Additionally, dermatological imaging often relies on dermoscopic features, which may not be directly applicable to forensic cases involving external trauma.

Similarities exist between forensic skin bruise detection and brain lesion analysis, particularly in the use of automated feature extraction and segmentation. DL techniques, especially CNNs, have been effectively leveraged for brain lesion segmentation and classification [14,21,26]. Although forensic skin bruise detection focuses on external injuries, methods used for brain lesion analysis could inform subsurface detection of skin bruising.

### 2.2. Computer Vision in Other Forensics Disciplines

AI-based image analysis has been increasingly utilized in other forensic fields, particularly in postmortem imaging and forensic pathology. A review [27] examined DL’s role in postmortem computed tomography (PMCT), a widely adopted forensic procedure. Given rising case volume and a shortage of postmortem radiology experts, DL offers a promising solution to enhance autopsy interpretation and workflow efficiency.

In forensic histopathology, a study [28] evaluated CNNs for myocardial infarction classification from histology slides. Comparing four common CNN architectures, InceptionResNet v2 achieved over 95% accuracy in distinguishing normal from acute and old myocardial infarctions. These findings suggest the DL’s applicability to forensic histopathological analysis. Similarly, DL has been applied to forensic firearm injury analysis, with models accurately classifying gunshot wound distances [29]. Collectively, these studies demonstrate successful integration of AI-driven image analysis into forensic workflows, reinforcing DL’s feasibility for forensic bruise detection.

### 2.3. Other Computer Vision AI Applications

Machine learning and computer vision are extensively applied in agriculture for identifying bruises and defects in fruits [30]. These models rely on spectral imaging, color analysis, and texture-based feature extraction [31], which bear relevance to forensic bruise detection, particularly in analyzing skin discoloration and injury severity. Techniques such as hyperspectral imaging and deep learning-based classification, commonly employed in fruit bruise detection [32], could be adapted to enhance bruise contrast in forensic imaging. However, forensic applications require adaptations, particularly in adjusting spectral feature selection to accommodate variations in human skin reflectance, vascularization, and bruise healing dynamics over time.

Computer vision and AI techniques have been extensively utilized in various engineering disciplines. For instance, in Non-Destructive Evaluation (NDE), particularly in civil engineering, they are applied to structural integrity assessment, defect detection, and surface condition monitoring. DL models, including CNNs and vision transformers, have been employed to identify structural defects, pavement distress, and pothole formation with high accuracy and efficiency [33,34,35]. These methods enhance traditional NDE by enabling automated, real-time anomaly detection, reducing reliance on manual inspections, and improving the predictive infrastructure maintenance.

The visual and morphological patterns analyzed in structural defect detection share conceptual similarities with forensic bruise detection. Both fields require precise localization, feature extraction, and pattern recognition to distinguish normal from anomalous conditions. Research has also explored the adaptation of Structural Health Monitoring (SHM) techniques for forensic bruise detection, applying lightweight DL models to improve forensic injury assessment [36].

## 3. Methodology

### 3.1. Dataset and Preprocessing

#### 3.1.1. Dataset Source and Description

The dataset utilized in this study was sourced from [7] and consists of high-resolution (6024 × 4022 pixels) images annotated for bruise detection. Annotations, specifying bruise presence and boundaries, were created using LabelBox [37] and verified through a multi-step validation process. Three independent annotators labeled bruises as “Yes” or “Unsure”, delineated boundaries, and provided pixel-wise segmentation where feasible. For consistency, an additional independent reviewer evaluated the annotations, flagging discrepancies for reassessment and correction according to a standardized protocol.

To consolidate multiple annotations, a union-based approach was applied, where the final annotation encompassed the entire area designated by any annotator, ensuring comprehensive coverage and minimizing the risk of omissions. This strategy prioritizes recall in forensic detection, where missed bruises carry higher operational risk than false positives requiring expert review. This approach also preserves weak, low-contrast bruises where annotator agreement is naturally imperfect, intentionally accepting increased overprediction as the cost of minimizing false negatives. Based on annotator agreement, the dataset was categorized into full consensus (all annotators labeled “Yes”, 12,802 images), partial consensus (a mix of “Yes” and “Unsure”, 1534 images), and complete uncertainty (all marked “Unsure”, 2488 images). To ensure high-confidence annotations for robust model training and evaluation, only the full-consensus subset was selected for this study.

The dataset includes images captured under varying lighting conditions and filter settings, comprising White light (W) images and alternate light source (ALS) images acquired with orange (O) and yellow (Y) filters. Other variations, which are not the focus of this study, were excluded from further analysis. The distribution of images across lighting conditions and data splits are summarized in Table 1.

RGB pixel statistics were analyzed to assess brightness and variability across imaging conditions. As shown in Table 2, the white light images exhibit higher mean values, particularly in the blue channel, indicating brighter images with greater standard deviation in red and blue, suggesting higher tonal variability. In contrast, the ALS dataset, including its filtered subsets, consistently displays lower mean values and reduced variability across all channels, reflecting a darker, more homogeneous distribution. These statistics provide a quantitative overview of dataset characteristics, offering insight into illumination-dependent variations in image appearance.

#### 3.1.2. Class Distribution and Bias Analysis

**Figure 1:**
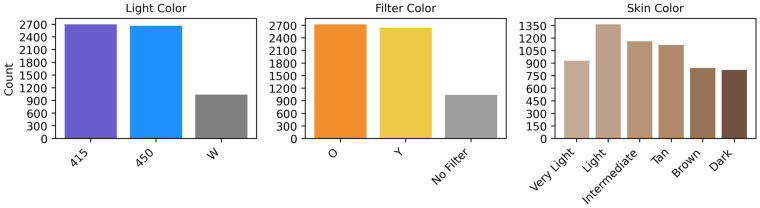
Distribution of full-consensus ALS+W dataset with respect to light color, filter color and skin tone.

Figure 1 illustrates the dataset distribution across light color, filter color, and skin color categories in the (ALS+W) dataset. The light color distribution shows that images captured under 415 nm and 450 nm wavelengths are well-represented, whereas White Light (W) images constitute a smaller proportion. The filter color distribution reveals a balanced presence of orange (O) and yellow (Y) filters, while a smaller subset of unfiltered (W) images remains. The skin color distribution highlights an overrepresentation of light color, with fewer images from dark and very light skin tones, suggesting potential biases in dataset composition.

To mitigate class imbalances, dataset balancing was applied at the entire dataset level before splitting into train/validation/test subsets. This process ensured proportional representation across light color, filter color, and skin color categories by grouping images based on these attributes and sampling equal instances from each group.

#### 3.1.3. Data Augmentation

On-the-fly data augmentation was applied exclusively to the training set during training to improve generalization and mitigate overfitting [15]. Both images and corresponding masks were resized to 224 × 224 pixels to match the input dimensions, ensuring compatibility with pre-trained feature extractors. Augmentation techniques included random horizontal and vertical flipping, affine transformations (cropping, rotation), and Gaussian noise injection to introduce variability. These transformations improved the model’s ability to recognize patterns across different orientations, scales, and lighting conditions, while also simulating real-world sensor noise. Table 3 summarizes the applied augmentation strategies and the corresponding parameters.

#### 3.1.4. Data Partitioning

The dataset was split into 70% training, 15% validation, and 15% testing. This partitioning was performed at the image level, as opposed to the subject level. While effective and common for general dataset management, this approach does not guarantee that images of the same physical injury are exclusively confined to a single split (e.g., training or testing). Consequently, there is a potential for some images of a given bruise to appear in both training and testing sets if that bruise was captured across multiple images, potentially leading to an inflated estimate of the model’s generalization performance on entirely novel injuries. This is discussed and considered in more detail in Section 3.4.4.

### 3.2. Object Detection Models

#### 3.2.1. Rationale for Model Selection

Modern object detection methods are categorized into two-stage (e.g., R-CNN series) and single-stage (e.g., SSD, YOLO) approaches. Two-stage methods first generate candidate bounding boxes before classification and refinement, prioritizing accuracy at the cost of computational efficiency. In contrast, single-stage methods predict object locations and categories in a single step, offering improved speed but generally lower accuracy [38,39].

This study evaluates widely recognized object detection models (Faster R-CNN, RetinaNet, and FCOS) selected based on their established performance, computational efficiency, and practical applicability [15]. The selection of these models was guided by key considerations:

Firstly, these models offer a well-documented balance between accuracy and computational efficiency. Faster R-CNN remains a highly effective two-stage detector, known for its high localization precision and robust performance across detection tasks [38]. FCOS and RetinaNet represent strong single-stage alternatives, with FCOS employing anchor-free detection and RetinaNet addressing class imbalance through focal loss.

Additionally, the widespread availability of these models in deep learning frameworks such as PyTorch facilitates reproducibility and seamless integration into existing pipelines, enhancing practical deployment and comparative analysis. The applied models have been extensively validated in the literature and across benchmark datasets, ensuring their reliability and broad adoption in both academic and industrial settings.

Each selected model aligns with specific forensic requirements: Faster R-CNN for high-precision detection, RetinaNet for handling class imbalance, and FCOS for its computational efficiency and speed advantages due to anchor-free detection.

#### 3.2.2. Faster R-CNN

Faster R-CNN [40] is a widely used two-stage detection model that balances accuracy and speed. It consists of a Region Proposal Network (RPN) and a detection network. The RPN generates object proposals from a shared convolutional feature map, reducing computational redundancy by utilizing the same backbone network for both proposal generation and classification [39]. The detection network then classifies proposals and refines bounding boxes [38,41].

In this study, an enhanced Faster R-CNN with a ResNet-50-FPN backbone implemented in PyTorch 2.8.0 with CUDA 12.6 is utilized as described in reference [42]. Additionally, to optimize performance on resource-constrained devices, MobileNetV3 [43] and its smaller variant, MobileNetV3 (320), are also considered. These models minimize memory and computational overhead by leveraging h-swish activation and eliminating computationally intensive operations [44,45].

#### 3.2.3. RetinaNet

RetinaNet [46], a one-stage detector, balances accuracy and efficiency by employing a dense set of predefined anchor boxes across multiple scales and aspect ratios. It utilizes ResNet with Feature Pyramid Network (FPN) as the backbone [47].

A key feature of RetinaNet is focal loss, which addresses class imbalance by reducing the weight of easily classified examples, focusing learning on harder misclassified instances [38]. This approach enhances detection robustness in datasets with underrepresented classes, a challenge relevant to the dataset used in this study.

#### 3.2.4. FCOS

Unlike anchor-based models (R-CNN, YOLO, RetinaNet), FCOS (Fully Convolutional One-Stage Object Detection) [48] eliminates the need for predefined anchor boxes, simplifying the detection pipeline and reducing computational complexity. Instead of anchors, FCOS predicts object locations at each spatial position on the feature maps.

A centerness score is used to filter out low-confidence detections by emphasizing the central regions of objects, improving boundary localization in dense scenes. This approach enhances detection performance in complex environments with significant variations in object scales.

### 3.3. Experimental Design and Training Protocol

#### 3.3.1. Experimental Objectives and Design

This study performs a structured evaluation of the three object-detection architectures to determine their suitability for forensic bruise detection. The evaluation has a twofold purpose: first, to quantify the relative performance of each architecture using standardized metrics and controlled preprocessing; and second, to characterize the conditions under which performance gains are robust versus artefactual (e.g., resulting from intra-injury image similarity instead of genuine generalization).

The experimental design adheres to principles of internal validity. All models were trained with the same preprocessing pipeline, augmentation policy, and ImageNet-derived backbone initializations to ensure observed differences reflect architectural and optimization effects rather than variations in data handling. Two hyperparameter tuning regimes were used: a fixed-hyperparameter regime for direct architectural comparison and a limited-budget tuning regime to normalize for optimization effort.

The experimental design involved controlled comparisons across pre-defined conditions relevant to forensic bruise detection. These included: same-condition evaluation (e.g., training and testing on ALS), cross-condition generalization (e.g., training on ALS and testing on W, with configurations detailed in Table 4), and threshold-sensitivity analysis. To address potential performance inflation from image-level partitioning, the evaluation considered both image-level and injury-level data splits, incorporating embedding similarity and seen/unseen injury comparisons (Section 3.4.4). Further assessments included analyzing the impact of dataset balancing (equalizing representation across skin tones, light colors, and filter settings) and a failure case analysis to identify performance determinants.

#### 3.3.2. Training Implementation and Hyperparameters

To ensure consistency in feature representation, input images were normalized using mean values [0.485, 0.456, 0.406] and standard deviation values [0.229, 0.224, 0.225], aligning with ImageNet pre-training. The number of classes was set to two (bruise and background).

Backbone weights were initially frozen to retain pre-trained feature representations from the COCO dataset, whereas the region proposal and classification heads were fine-tuned. An unfrozen configuration was also tested to evaluate its impact on performance.

Hyperparameter optimization was conducted to balance computational efficiency and model performance. The initial learning rate was set to 1 × 10^−3^, with step decay applied when the validation loss plateaued. Alternative learning rates were examined to evaluate their effect on stability and convergence. The batch size was fixed at 16, constrained by hardware limitations (Nvidia A100—40GB GPUs, NVIDIA Corporation, Santa Clara, CA, USA), as larger batch sizes resulted in memory overflow, particularly in models with higher parameter counts such as RetinaNet.

The Adam optimizer was selected for its adaptive moment estimation, which adjusts learning rates per parameter, improving convergence in non-stationary loss landscapes. However, Adam’s tendency to converge quickly but risk suboptimal generalization necessitated comparison with SGD with momentum. Momentum helps stabilize updates by accumulating gradient history, reducing oscillations, and improving localization accuracy. This comparison provided insights into the impact of adaptive vs. momentum-based optimization on detection robustness and convergence behavior.

#### 3.3.3. Cross-Condition Generalization Strategies for Training

Model performance is influenced by variations in illumination, necessitating generalization strategies to enhance robustness across different lighting conditions. The dataset includes White Light (W) and Alternate Light Source (ALS) images, each presenting distinct challenges. W images are more abundant but exhibit lower bruise contrast, complicating annotation, whereas ALS images enhance visibility but are less accessible and require specialized acquisition.

Achieving a balanced distribution of both types is essential for model deployment and also improving detection reliability under diverse conditions. Empirical studies on object detection under varying lighting conditions indicate that incorporating images captured at different times of the day improves model generalization, leading to enhanced performance across both high- and low-light scenarios [49]. This highlights the importance of balanced training data and the effectiveness of mixed training in optimizing deep neural network performance.

Two mixed training approaches were evaluated. The first was a uniform mix, where W and ALS images were equally represented in each batch to expose the model to varied conditions. The second was a varied mix, where the ratio of W-to-ALS images was systematically adjusted (100/0, 75/25, 50/50, 25/75, and 0/100) to identify the optimal data composition for generalization. Total dataset size was held constant across these experiments to ensure observed performance changes were attributable to data composition alone.

In addition to data-level strategies, training was optimized through algorithmic and heuristic methods. Algorithmic techniques included focal loss, which prioritizes hard-to-classify examples. Heuristic approaches included targeted retraining on misclassified or ambiguous images to reinforce learning on challenging instances.

### 3.4. Evaluation Protocol

#### 3.4.1. Core Performance Metrics

Model performance was evaluated using standard object detection metrics. Intersection over Union (IoU), or the Jaccard index, was used to measure the overlap between predicted and ground-truth bounding boxes [39]. Based on a specified IoU threshold, detections were classified as True Positives (TP), False Positives (FP), or False Negatives (FN).

These classifications were used to calculate Precision (the fraction of correct predictions among all predictions) and Recall (the fraction of ground-truth objects correctly detected). The F1 score was used as the harmonic mean of precision and recall. Per-class performance was measured by Average Precision (AP), the area under the precision-recall curve.

Two AP-based metrics are reported throughout this study. First, mAP@0.5 is computed following COCO conventions with IoU fixed at 0.5, to facilitate comparison with the broader detection literature. Second, a multi-threshold average AP is computed as the mean of AP values evaluated across IoU thresholds {0.1, 0.3, 0.5, 0.7, 0.9} and confidence thresholds {0.1, 0.3, 0.5, 0.7, 0.9}. This custom metric captures model stability across the full operational threshold space, reflecting the deployment reality that forensic applications require case-specific threshold tuning rather than a single canonical operating point.

To refine detection reliability, confidence thresholds were applied to eliminate low-confidence predictions, ensuring only high-certainty detections were considered. Non-Maximum Suppression (NMS) was implemented to remove redundant bounding boxes, thereby optimizing the trade-off between precision and recall.

#### 3.4.2. Detection (Confidence) Thresholding and IoU Variability Analysis

Confidence threshold selection directly affects precision-recall trade-offs in object detection [50,51]. Lower thresholds increase recall but introduce more false positives, whereas higher thresholds improve precision at the risk of missing detections. This is particularly critical in high-stakes applications such as forensic analysis and medical diagnostics, where incorrect detections have significant consequences. To systematically evaluate these trade-offs, the same confidence thresholds and IoU thresholds described in Section 3.4.1 were tested.

#### 3.4.3. Image-Specific and Failure Analysis

**Figure 2:**
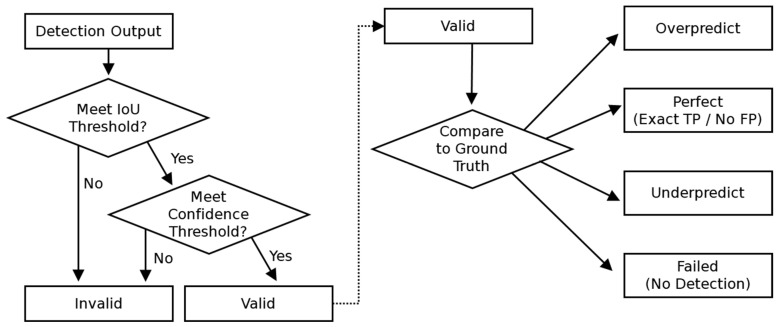
Workflow for prediction validity and status analysis.

Model evaluation was conducted to systematically assess detection accuracy and reliability through analysis of three primary aspects: (1) Prediction Validity, (2) Status, and (3) Variance. Prediction validity was determined based on predefined IoU and confidence thresholds, with detections classified as either Valid or Invalid (Figure 2, left).

To provide a granular evaluation beyond binary classification (match/non-match approach), prediction status was categorized into four classes (Figure 2, right): Perfect, Underpredict, Overpredict, and Failed. Perfect predictions matched the ground truth with no false positives. Underprediction occurred when the number of true positives was lower than expected, whereas overprediction indicated excessive bounding box detections, introducing false positives. Cases where a ground-truth object had no corresponding model prediction meeting the predefined IoU and confidence thresholds were classified as Failed.

To assess confidence stability, prediction variance was quantified using the standard deviation of confidence scores. Predictions with a variance below the 75th percentile threshold were labeled Consistent, whereas those exceeding this threshold were considered Inconsistent, indicating higher uncertainty and potential unreliability.

A structured evaluation approach was used to analyze model performance across different image attributes, incorporating skin tone, lighting conditions, and filter type. A baseline IoU and confidence threshold (0.5, 0.5) was applied to extract metadata and categorize images based on prediction validity, status, and variance. Visualizations were generated to examine the distribution of prediction classifications and variance levels across models. Additionally, the impact of key imaging attributes, such as lighting color and skin tone, was analyzed to assess their influence on detection accuracy.

The study also investigated cases where detections were consistently predicted across different models, identifying common factors contributing to detection success and variations in model performance under different conditions. Challenging cases, defined as instances where the model exhibited inconsistencies, misclassifications, or failed detections, were further examined. These cases typically resulted from low contrast, poor illumination, noise, artifacts, or imaging condition variations, making them critical for assessing model robustness. Challenging cases were classified using the same Failed, Underpredict, Overpredict, and Inconsistent categories, ensuring consistency in evaluation.

To assess image degradation and its impact on detection performance, No-Reference Image Quality Assessment (NR-IQA) metrics were selected based on their applicability to natural and medical imaging. BRISQUE [52] quantifies deviations from natural scene statistics, capturing perceptual distortions such as blurring and compression artifacts [23]. NIQE [53] evaluates image quality by modeling statistical deviations from pristine images and has been applied to skin image quality assessment [54]. IL-NIQE [55], an opinion-unaware method, applies multivariate Gaussian modeling to assess quality without requiring subjective scores, improving generalization across unknown distortions. LIQE [56] incorporates multitask learning across blind IQA, scene classification, and distortion-type identification, enhancing its robustness in diverse imaging conditions. These metrics collectively provide a structured framework for evaluating image quality and its effect on detection performance.

To rigorously assess whether dataset balancing produces statistically significant and clinically meaningful differences in performance across skin-tone subgroups, formal hypothesis testing was conducted. For each skin tone and outcome (Failure Rate, Overprediction Rate, and Localization Variance Rate), 95% Wilson score confidence intervals were computed and two-sided Fisher’s exact tests were performed comparing unbalanced vs. balanced proportions.

#### 3.4.4. Analysis of Performance Dependency on Data Partitioning

To investigate the effect of the image-level data partitioning, two specific analyses were conducted. The first was an Embedding Similarity Analysis (ESA). Feature embeddings were extracted for all images using each model’s pre-trained backbone.

Visual similarity was quantified as the Euclidean distance from each test image to its nearest neighbor in the training set’s embedding space. Prior to distance computation, embedding features were standardized using z-score normalization based on training-set statistics to ensure that dimensions with larger variance did not disproportionately influence similarity calculations. Euclidean distance was selected as a simple and interpretable nearest-neighbor similarity metric because the analysis aimed to quantify relative train–test proximity rather than optimize feature similarity through metric learning, and Euclidean distance is commonly used as a baseline for nearest-neighbor similarity analysis in medical machine learning applications [57,58]. Test images were then binned into quantiles based on this similarity metric: Top 20% Most Similar (n = 194), Middle 60% (n = 580), and Bottom 20% Least Similar (n = 194).

The second was a comparison between seen and unseen injuries. Model performance was evaluated on two distinct, balanced test sets: one containing images of “seen injuries” (injuries that also had other images present in the training set) and another containing images of “unseen injuries” (injuries with no corresponding instances in the training set).

## 4. Results and Discussion

### 4.1. Analysis of Object Detection Models

#### 4.1.1. Performance Evaluation of Faster R-CNN Variants

This section evaluates the performance of Faster R-CNN with different backbones (MobileNet 320, MobileNet, and ResNet-50) across various optimization strategies (SGD and ADAM) and learning rates.

Faster R-CNN MobileNet 320 demonstrated unstable loss convergence, particularly with SGD, where loss fluctuations persisted across different learning rates. Even with ADAM, loss oscillations were evident, especially at higher learning rates (~0.001), indicating difficulties in stabilizing gradient updates. The lightweight nature of MobileNet 320, optimized for computational efficiency, may lack sufficient feature extraction capacity for this task, leading to training instability. MobileNet (standard) exhibited improved performance, with higher mAP and recall compared to MobileNet 320, though loss fluctuations were still observed, suggesting sensitivity to learning rate selection. ResNet-50, trained with ADAM at 0.001, achieved highly stable convergence, with the highest mAP and recall, confirming its superior feature extraction capabilities.

**Figure 3:**
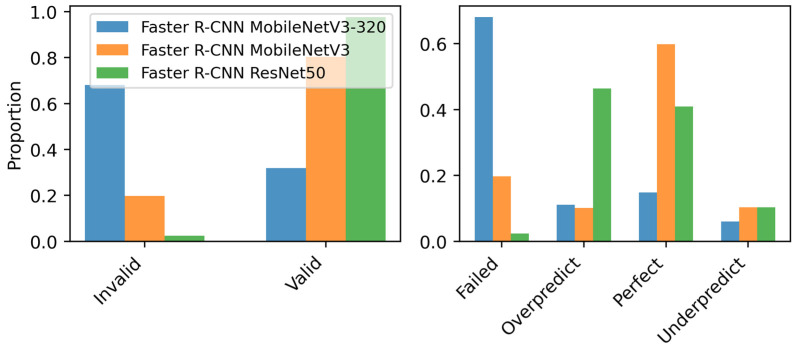
(**Left**) Prediction validity across Faster R-CNN models. (**Right**) Distribution of failed, overpredicted, perfect, and underpredicted detections.

The following analysis also incorporates the failure-case metrics (prediction validity, status, and variance) defined in Section 3.4.3 to provide deeper insight into model reliability. Figure 3 (left) shows that ResNet-50 achieved the largest proportion of valid detections, whereas MobileNet 320 had the largest rate of invalid predictions, highlighting its lower reliability. The prediction status analysis in Figure 3 (right) reveals that MobileNet 320 exhibits the highest proportion of failed detections, confirming its instability and reduced sensitivity. ResNet-50, despite superior performance, shows a notable overprediction tendency, leading to increased false positives. MobileNet, while more balanced in terms of precision and recall, did not surpass ResNet-50 in overall detection performance.

**Figure 4:**
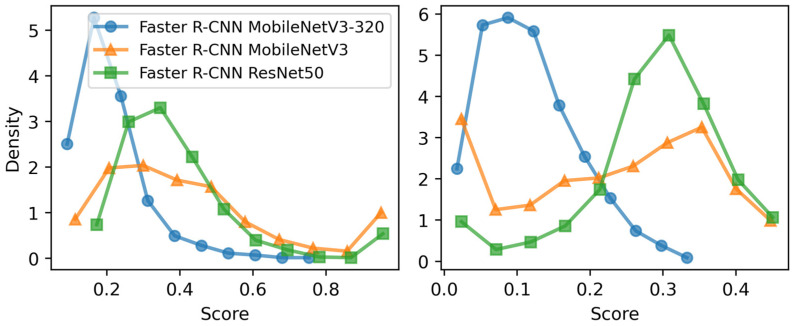
Confidence score distribution across Faster R-CNN backbones for all predicted bounding boxes. (**Left**) Mean confidence scores, indicating overall model confidence in detections. (**Right**) Standard deviation of confidence scores, reflecting prediction consistency and uncertainty.

The confidence score analysis on the test dataset reveals distinct trends among the models (Figure 4). MobileNet 320 consistently produced low-confidence predictions (~0.2) with minimal variance (~0.1), often resulting in detections being filtered out despite accurate localization. In contrast, ResNet-50 exhibited higher confidence scores (~0.45) with greater variability (~0.3), reflecting stronger classification certainty. MobileNet, positioned between the two, demonstrated a more balanced confidence distribution. Overall, ResNet-50 achieved the highest observed confidence in predictions, enhancing classification reliability, whereas MobileNet 320’s low average confidence indicates greater uncertainty in its detections.

#### 4.1.2. Analysis of Detection Performance and Reliability Across Models

The statistical distribution of confidence scores highlights distinct model behavior patterns. FCOS achieves the highest observed mean confidence score, approximately 20% higher than RetinaNet and 80% higher than Faster R-CNN, indicating stronger classification confidence. This suggests that FCOS provides more reliable predictions, making it a preferable choice for further evaluations. Additionally, the standard deviation analysis reveals that FCOS exhibits very low confidence variability (~0.05), compared to RetinaNet (~0.15) and Faster R-CNN (~0.25). The lower variance in FCOS predictions suggests greater consistency in confidence scores, reducing uncertainty and enhancing reliability in bruise detection.

The analysis of TPs and FPs reveals key performance differences across the models. FCOS achieves the largest number of TPs, closely matching RetinaNet, while both significantly outperform Faster R-CNN, which detects approximately three times fewer TPs. In terms of FPs, Faster R-CNN produces the largest number, followed by FCOS, with RetinaNet exhibiting the lowest FP count. This suggests that while Faster R-CNN is less effective at detecting true positives, it also generates more false positives, indicating potential issues with bounding box refinement or classification confidence. FCOS, on the other hand, balances a higher TP rate with a moderate FP count, making it a strong candidate for bruise detection in this context.

**Figure 5:**
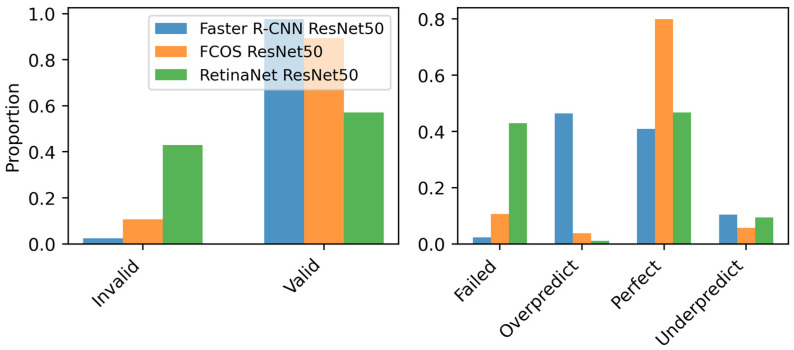
Prediction validity and error distribution for Faster R-CNN, FCOS, and RetinaNet with ResNet-50 backbone. (**Left**) Valid vs. invalid detections. (**Right**) Distribution of failed, overpredicted, perfect, and underpredicted detections.

Prediction validity and error distribution (Figure 5) highlight the reliability of each model. FCOS achieves a strong balance between valid detections and perfect predictions while maintaining a lower failure rate compared to RetinaNet. Faster R-CNN exhibits the highest valid detection rate but at the cost of increased overpredictions. RetinaNet shows a higher failure rate, indicating potential instability. Overall, FCOS demonstrates a favorable trade-off between detection accuracy and error minimization.

### 4.2. Model Performance and Cross-Dataset Generalization

The evaluation of model performance across different train–test configurations, summarized in Table 5, provides insights into detection accuracy and generalization capabilities under varying illumination conditions. Two metrics are reported: a multi-threshold average AP (incorporating all IoU and confidence threshold combinations) to capture operational robustness, and mAP@0.5 for comparison with standard benchmarks.

When trained and tested on the same dataset, FCOS consistently outperforms Faster R-CNN and RetinaNet, achieving the highest observed AP across all conditions. This suggests that its architecture is better suited for bruise detection under both standard and alternate light conditions. This ranking is consistent across both metrics, with mAP@0.5 values further confirming FCOS superiority. RetinaNet performs comparably to Faster R-CNN in most cases, with marginally higher AP scores.

Cross-dataset evaluation reveals a decline in model performance when tested on a dataset different from its training distribution. As shown in Table 5, FCOS demonstrates superior cross-dataset generalization, particularly in ALS conditions, maintaining relatively high AP even when trained on ALS+W. Generally, models trained on ALS+W and tested on either W or ALS exhibit lower APs compared to those trained and tested on a single condition, except for FCOS, which achieves a higher AP in W testing conditions when trained on ALS+W than when trained solely on W. However, since the primary objective is to develop a model that generalizes well to White Lighting conditions, further analysis is conducted to determine the effective ALS-to-W ratio in the training dataset. This ensures that the model leverages the advantages of ALS training without overfitting to a specific illumination condition, maintaining strong predictive performance on W images.

### 4.3. Influence of W/ALS Ratio on Model Training Performance

The effect of varying the W/ALS ratio on FCOS model performance was evaluated using AP, cumulative true positives (CUM TP), cumulative false positives (CUM FP), Precision, Recall, and F1-score (Table 6). Both the multi-threshold AP and mAP@0.5 peak at 0/100 (pure ALS) and decline as W ratio increases, reaching the minimum at 100/0 (pure W), indicating that ALS data contributes more effectively to detection performance, whereas higher W ratios degrade accuracy. CUM TP peaks at 25/75 (69 TP) but decreases beyond 50% W, whereas CUM FP increases significantly with higher W ratios, leading to lower precision. Precision is at its highest at 0/100 and decreases with more W data, whereas recall peaks at 25/75, suggesting a slight improvement in detection sensitivity at this ratio. However, both precision and recall drop at higher W ratios, resulting in the lowest F1-score at 100/0, reflecting poor performance when relying solely on W data. The results indicate that ALS data enhances AP, precision, and F1-score, while higher W ratios increase false positives and reduce model reliability. A small fraction of W data (25%) improves recall but does not enhance AP or precision, offering limited benefit. The findings suggest that ALS-dominant datasets yield better performance, and higher W ratios consistently degrade accuracy, emphasizing the need to prioritize ALS data for optimal model training.

The observed performance advantage of ALS-dominant training is consistent with the established optical properties of 415 nm and 450 nm illumination. Hemoglobin exhibits a sharp absorption peak at approximately 415 nm, and bilirubin absorbs broadly around 460 nm; these wavelength-specific absorptions produce inherently higher bruise-to-background contrast than broad-spectrum white light that emits across the full visible spectrum without isolating individual chromophore responses [7,10,59]. This optical advantage is reflected empirically in the lower RGB variance observed in ALS images (Table 2), which in turn facilitates more reliable feature extraction by the detection backbone.

### 4.4. Effect of Confidence and IoU Threshold Variations

**Figure 6:**
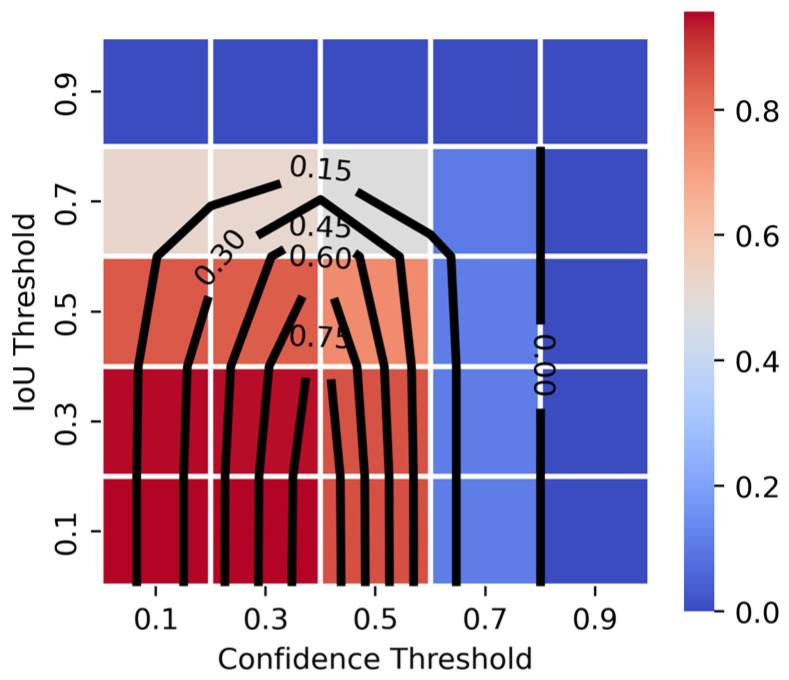
AP heatmap with F1 score contours for the W0 ALS100 dataset (FCOS).

The effect of confidence and IoU thresholds on model performance was evaluated using various metrics across different threshold configurations (Table 7). Figure 6 provides a visual representation of the optimal confidence and IoU thresholds for maximizing AP and F1-score.

AP is highest at lower confidence thresholds (0.1–0.3) and declines as confidence increases, reaching its lowest values at 0.7 and above. Lower IoU thresholds (0.1–0.3) maintain higher AP, indicating that the model performs better under lenient localization constraints. However, AP drops significantly when both confidence and IoU thresholds are high, suggesting that stricter filtering eliminates many detections.

Precision improves at higher confidence thresholds due to reduced false positives but at the expense of recall, which decreases significantly. The inverse relationship between precision and recall is evident, particularly at high IoU thresholds, where stricter localization criteria further reduce recall. The optimal balance is observed at moderate confidence thresholds (~0.3–0.5), where both recall and precision remain stable.

F1-score follows a similar trend, peaking at a confidence threshold of 0.5. Higher confidence settings improve precision but lead to missed detections, while lower thresholds enhance recall but increase false positives. Notably, confidence thresholds above 0.7 result in almost no detections, and IoU thresholds greater than 0.5 significantly degrade performance.

In Figure 6, the heatmap represents AP, while contours depict F1-score. While AP is highest at confidence = 0.1 to 0.3, F1-score peaks at confidence = 0.5. The confidence = 0.3/IoU = 0.3 operating point represents a practical balance: it maintains near-peak AP while achieving substantially better precision-recall balance than the lowest confidence thresholds (F1 = 0.036 at confidence = 0.1/IoU = 0.1 vs. F1 = 0.387 at confidence = 0.3/IoU = 0.3), and avoids the severe recall collapse observed at confidence ≥ 0.7. This trend is consistent across different W/ALS ratios and configurations. However, optimal threshold values are model-dependent: Faster R-CNN achieves peak performance at a confidence threshold of 0.1, whereas RetinaNet exhibits behavior similar to FCOS.

Optimal threshold selection ultimately depends on the forensic application’s risk profile: lower thresholds prioritize sensitivity (recall), while higher thresholds prioritize specificity (precision).

### 4.5. Impact of Dataset Balancing on Model Performance

#### 4.5.1. Overall Model Performance

**Figure 7:**
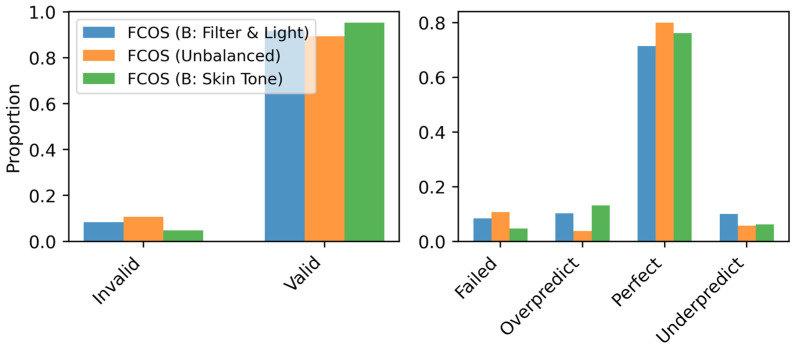
(**Left**) Prediction validity across FCOS models trained on the original and balanced datasets. (**Right**) Distribution of failed, overpredicted, perfect, and underpredicted detections.

**Figure 8:**
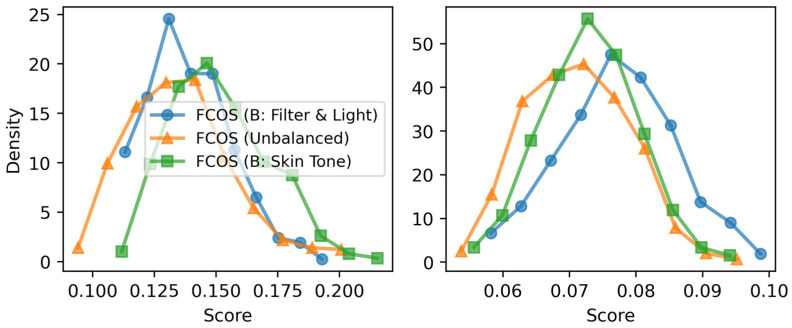
Confidence score distribution for FCOS across balanced and unbalanced datasets. (**Left**) Mean confidence scores. (**Right**) Standard deviation of confidence scores.

The effect of dataset balancing on FCOS performance was evaluated across three configurations: the original (ALS+W) dataset, a light/filter-balanced dataset, and a skin-balanced dataset. As shown in Figure 7 and Figure 8, balancing influences prediction validity, error characteristics, and confidence scores, with distinct effects depending on the balancing strategy.

Prediction validity analysis (Figure 7, left) indicates that balancing enhances detection reliability, increasing the valid prediction rate to 95.29% for the skin-balanced dataset and 91.67% for the light/filter-balanced dataset, compared to 89.36% for the original dataset. The original dataset exhibits a higher invalid detection rate, likely due to class imbalances limiting generalization. This suggests that balancing improves robustness by reducing underrepresented cases.

Prediction status distributions (Figure 7, right) show that balancing reduces failed detections, while all configurations maintain a high proportion of perfect detections. However, overprediction increases in the balanced datasets, leading to a small drop in perfect prediction rates (from 89.48% in the original dataset to 79.84% and 77.86% in the skin-balanced and light/filter-balanced datasets, respectively). This suggests that balancing increases the model’s sensitivity to detecting objects, potentially at the cost of an increase in false positives.

The confidence score distributions further explain these trends. The mean confidence score distributions (Figure 8, left) show that the light/filter-balanced dataset shifts scores toward higher values, indicating greater prediction certainty. The skin-balanced dataset follows a similar trend but exhibits a broader distribution, suggesting higher variability in confidence scores. The standard deviation analysis (Figure 8, right) shows comparable variance across all configurations, confirming that dataset balancing does not introduce excessive prediction uncertainty.

#### 4.5.2. Skin Tone Fairness

While dataset balancing improved overall prediction reliability, its effectiveness varied across skin tones. In the context of detection fairness, defined as the model’s ability to maintain consistent performance across subgroups, balancing seeks to mitigate biases in the original dataset and promote more equitable predictions. By improving class representation, balancing has the potential to enhance generalization across skin tones and lighting conditions, but its impact on specific prediction categories remains uncertain. To assess whether balancing achieves this goal and whether observed differences are statistically significant and not artifacts of small-sample effects, we conducted formal statistical testing per skin-tone subgroup.

Table 8 compares the performance of the Skin-balanced (Sb), Light/Filter-balanced (LFb), and Unbalanced (U) datasets across Failed, Overpredict, Perfect, and Underpredict classifications. Table 9 and Table 10 provide statistical comparisons for the two primary outcomes of interest (Failure Rate and Overprediction Rate), including 95% Wilson score confidence intervals, sample sizes, and Fisher’s exact test *p*-values.

To rigorously assess whether skin-balanced training reduces failure rates across skin-tone subgroups, we compared U vs. Sb failure proportions for each skin tone using Fisher’s exact test, with 95% Wilson score confidence intervals (Table 9). Sample sizes per skin tone ranged from n = 120 to n = 188.

Skin balancing reduced failed predictions across all skin tones, according to Table 8. However, the training produced statistically significant reductions in failure rates only for the darker-skinned subgroups (Brown, Dark, and Intermediate) while the lighter skin tones showed no significant reduction (Table 9). This pattern indicates that balancing disproportionately benefits detection in darker skin tones, where baseline failure rates were higher, yielding a fairness gain that directly addresses detection disparity. Although lighter tones also showed reductions, the effect was less pronounced. Despite the underrepresentation of very light skin tones, failure-rate reductions remained modest, indicating that balancing primarily benefits darker tones rather than merely addressing class underrepresentation.

While skin-balanced training reduced failure rates in darker tones, it introduced a substantial and counterbalancing increase in overprediction rates (Table 10). Across five of six skin tones, the increase was statistically significant. Only Intermediate showed no significant increase. The magnitude of these increases is substantial: five of six skin tones experienced absolute increases of 8–15 percentage points in false positives. These increases are not artifacts of small samples and represent a genuine trade-off between sensitivity (catching missed bruises) and specificity (avoiding false alarms).

Contrary to expectations that balancing might improve confidence consistency, localization variance rates remained stable across all skin-tone subgroups (all pairwise comparisons: *p* > 0.05). This indicates that the increase in overprediction arises not from confidence uncertainty (Figure 8), but from multiple overlapping bounding boxes assigned to the same bruise (Section 4.6.2, Figure 10b). Addressing this issue therefore requires refined non-maximum suppression and localization-weighted loss functions, not confidence-threshold adjustments.

The effect on the Perfect class was mixed. Lighter skin tones maintain high accuracy with minor reductions, whereas darker tones show little improvement or slight declines. This aligns with the rise in overprediction, indicating the model detects bruises in darker skin but struggles with precise localization. The tendency to identify but misplace bruises results in spatial inconsistencies in bounding box placement and overestimation.

Underprediction errors increased slightly for lighter and intermediate tones, suggesting a shift in classification tendencies rather than uniform improvement. However, darker tones saw a reduction in underprediction errors, indicating that balancing makes the model less likely to overlook bruises in darker-skinned inputs.

The statistical analysis reveals a fundamental tension in dataset balancing for this forensic application: improvements in detection equity (lower failure rates in darker skin tones) are achieved at the cost of reduced specificity (higher false-positive rates across most groups). This trade-off is statistically significant and not an artifact of sample size, with large effect sizes (8–15 pp increases in overprediction). The clinical or forensic acceptability of this exchange depends on application priorities: systems prioritizing sensitivity (avoiding missed lesions, critical for victim documentation) may accept higher false-positive rates; systems prioritizing specificity may require alternative balancing or regularization strategies.

Unlike skin-balanced training, which produced large but opposing effects on failure and overprediction rates, the LFb approach resulted in more modest changes. Although it slightly reduced failure rates compared to the unbalanced dataset (Table 8), the reductions were smaller and less consistent across skin tones than the Sb approach. Simultaneously, LFb increased overprediction frequency while decreasing perfect predictions across all skin tones, suggesting a decline in localization precision without proportionate gains in detection coverage. The combination of modest failure-rate improvements and degraded precision indicates that LFb is less effective than Sb in addressing dataset bias while maintaining specificity.

### 4.6. Error Analysis and Performance Influences

#### 4.6.1. Influence of Dataset Attributes on Detection Outcomes

**Figure 9:**
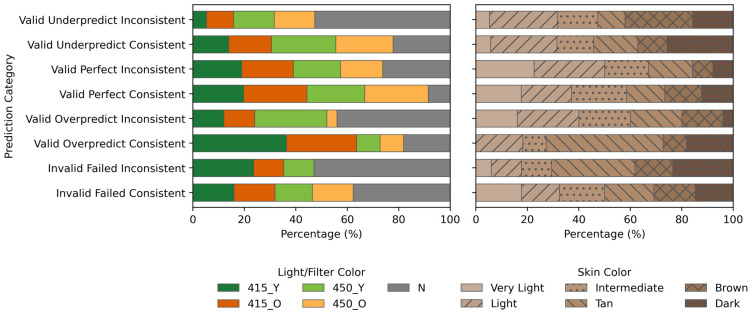
Normalized percentage distributions of FCOS detections across dataset attributes. (**Left**) Light/Filter Color Distribution across different prediction categories. (**Right**) Skin Color Distribution highlighting variations in detection reliability across different skin tones. Each bar represents the proportion of each attribute within its respective detection category.

To analyze how FCOS predictions are influenced by dataset attributes, the distribution of detections across light/filter colors and skin colors was analyzed (Figure 9). The analysis considered both absolute counts and normalized percentages within each category, ensuring that the results reflect relative representation rather than raw frequency disparities. This approach highlights potential biases or model sensitivities to specific imaging conditions. The distribution percentages for each attribute were computed by (count of attribute A in category C) over (total count in category C). This represents the proportion of attribute A (e.g., a specific light condition or skin tone) within the detection category C (e.g., valid, failed, perfect). Consequently, each bar within the plot illustrates the percentage composition of each attribute relative to its specific detection category, rather than across the entire dataset.

The analysis of optimal detection outcomes (Valid Perfect Consistent) within the light/filter distribution indicated a higher representation under 450 nm and 415 nm conditions. Conversely, the poorest detection outcomes (Invalid Failed Inconsistent) are predominantly associated with White Light (W), followed by the 415 nm condition, suggesting increased prediction instability under these conditions.

A similar trend is observed in the skin color distribution. In the best-case category (Valid Perfect Consistent), skin tones are more uniformly represented, but Tan and Dark skin tones appear underrepresented. This suggests that the model performs relatively well across skin tones but may have reduced sensitivity to darker tones. In contrast, the worst-case scenario (Invalid Failed Inconsistent) shows a higher proportion of darker skin tones, indicating that misclassifications and inconsistent detections occur more frequently for these categories.

A percentage-based evaluation of detection outcomes across different lighting and filter conditions was conducted to complement previous analyses and refine model performance insights (Table 11). 450 nm lighting exhibits greater stability, with fewer failed detections compared to 415 nm, which introduces higher model instability, likely due to increased contrast variations. Overprediction rates are higher under 415 nm and yellow (Y) filters, particularly in inconsistent predictions, indicating a greater tendency for false positives. In contrast, 450 nm with orange (O) filters demonstrates reduced overpredictions, suggesting better specificity in bruise detection. Perfect prediction analysis further supports the advantage of orange filters, which result in more stable and confident detections compared to yellow filters. However, 415 nm lighting conditions introduce greater variability in perfect predictions, reinforcing its tendency toward inconsistent detection performance. Underpredictions remain relatively stable across all conditions but are slightly higher under 450 nm, indicating a conservative detection approach in this setting. Overall, 450 nm lighting combined with orange filters yields the most reliable performance, with lower failure rates and improved detection stability, making it the preferred configuration for bruise detection.

A similar analysis across different skin tones is presented in Table 12. The results indicate that darker skin tones exhibit higher failure rates, while intermediate skin tones achieve the highest perfect prediction rates, suggesting greater model stability and accuracy in this category. The high proportion of inconsistencies in the perfect prediction category suggests variability in model confidence and potential sensitivity to illumination or contrast differences across skin tones. Underprediction rates remain relatively stable, though dark skin tones show slightly higher consistent underpredictions, indicating a more conservative detection approach in this category. Overall, the model exhibits performance variability across skin tones, with intermediate skin tones yielding the most stable predictions, while very light and dark skin tones show higher inconsistencies, likely due to model sensitivity to variations in contrast and pigmentation.

#### 4.6.2. Failure Cases and Model Limitations

Error analysis is essential for understanding model performance, particularly in instances of failed detections, underprediction, and overprediction. Several factors contribute to detection failures and underprediction, including low contrast (faded bruises), label inaccuracies, blurriness, camera shift, and shadows. These issues introduce inconsistencies in detection, affecting model robustness. A combination of quantitative and qualitative analyses was conducted to systematically assess these failure cases.

To identify underlying patterns, multiple features were analyzed, including image quality metrics, skin color, light color, and filter color across all images. Classifiers were trained to distinguish between failed, overpredicted, underpredicted, and correctly predicted cases (Table 13). The impact of dataset composition, balancing strategies, and bruise age distribution variations was also examined. Additionally, K-Means clustering was applied to group similar images based on extracted features. While clustering performance was limited, its integration with Permutation Feature Importance (PFI) analysis provided valuable insights into feature influence on prediction outcomes.

The results indicate that lighting conditions, image quality, bruise age, and skin color significantly affect detection performance. Images captured under White Lighting conditions exhibited shifts in prediction distribution, highlighting its impact on model reliability. Variations in shadows, blurriness, noise, and image clarity emerged as primary factors contributing to inconsistencies in detection. Older bruises posed a greater challenge due to fading and reduced contrast, leading to inconsistencies even among human annotators. Although skin color influenced detection performance, bruise age and image quality had a more substantial effect.

**Figure 10:**
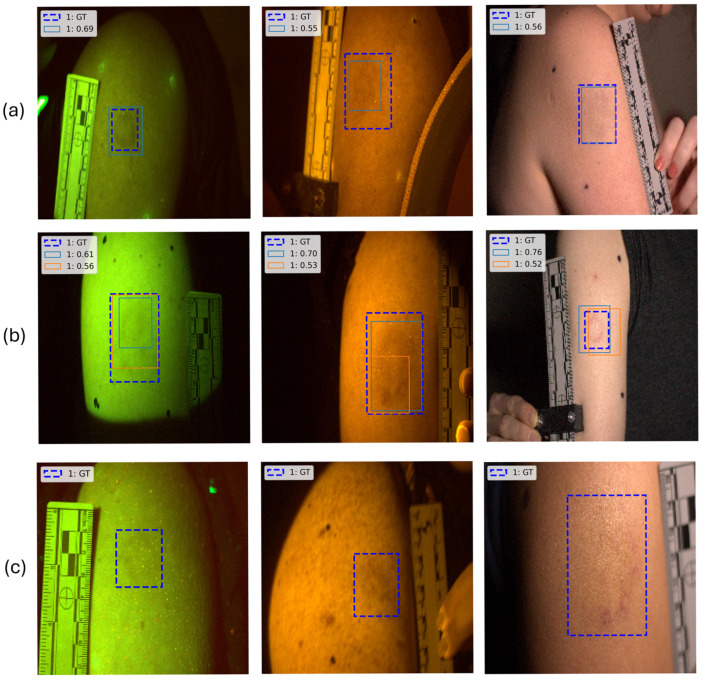
Detection outcomes: (**a**) True positives, (**b**) False positives due to overprediction, and (**c**) False negatives due to missed detections.

Figure 10 illustrates different detection outcomes, including true positive cases where bruises were correctly detected, false positives, overprediction and false negatives, where actual bruises were missed due to low contrast or image quality limitations. False positives were primarily caused by overlapping detections. As shown in Figure 10b, multiple bounding boxes were assigned to the same bruise with varying confidence scores, leading to redundant detections and reduced precision. Mitigating this issue requires penalizing redundant detections during training and refining the Non-Maximum Suppression (NMS) technique to merge overlapping bounding boxes, rather than simply increasing the confidence threshold globally, which risks significant recall loss.

### 4.7. Impact of Data Partitioning on Generalization

The image-level partitioning used for the primary analysis resulted in subject injury overlap between training and test sets. In the ALS+W dataset, 100% of test images belonged to injuries that also had other instances present in the training set. This overlap, combined with controlled acquisition conditions, may yield performance estimates that overstate generalization to unseen injuries.

**Figure 11:**
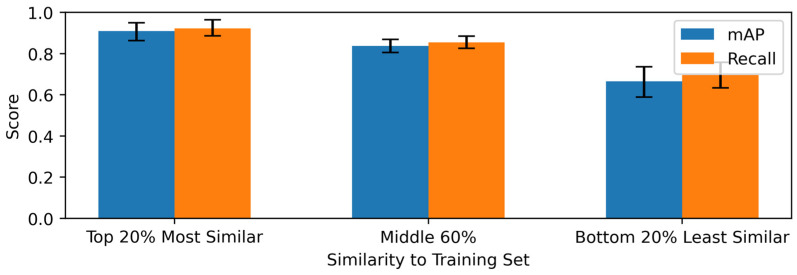
Model performance (mAP and Recall) stratified by test-to-train visual similarity quantiles at 0.5 IoU and 0.5 confidence. Error bars represent 95% confidence intervals obtained via bootstrapping.

**Figure 12:**
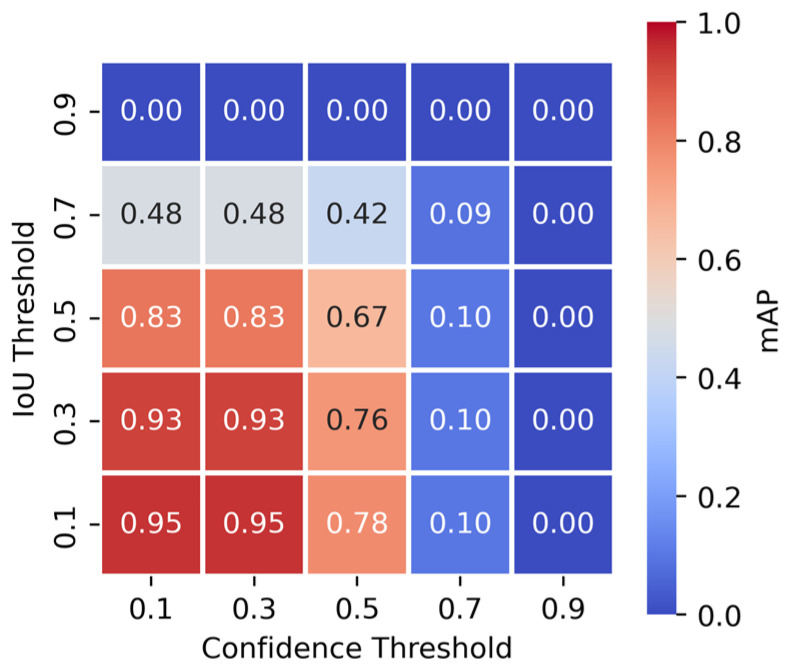
mAP heatmap for the Bottom 20% Least Similar test quantile across IoU and confidence thresholds.

Performance (mAP and Recall) was evaluated over sweeps of IoU and confidence thresholds. Figure 11 presents results at a fixed operating point (0.5 IoU, 0.5 confidence), revealing a gradient: mAP decreased from 0.91 (95% CI: [0.86, 0.95]) in the Top 20% bin to 0.84 (95% CI: [0.80, 0.87]) in the Middle 60%, and 0.67 (95% CI: [0.59, 0.74]) in the Bottom 20% (a 26% relative decline). Recall followed a similar pattern, declining from 0.92 to 0.70. Heatmaps (Figure 12) indicate sensitivity to thresholds in the least-similar bin: mAP exceeded 0.80 at lenient settings (e.g., 0.83 at 0.5 IoU/0.1 confidence) but declined sharply at stricter ones (e.g., <0.10 at 0.7 confidence). Comparable threshold dependencies were observed across bins.

**Figure 13:**
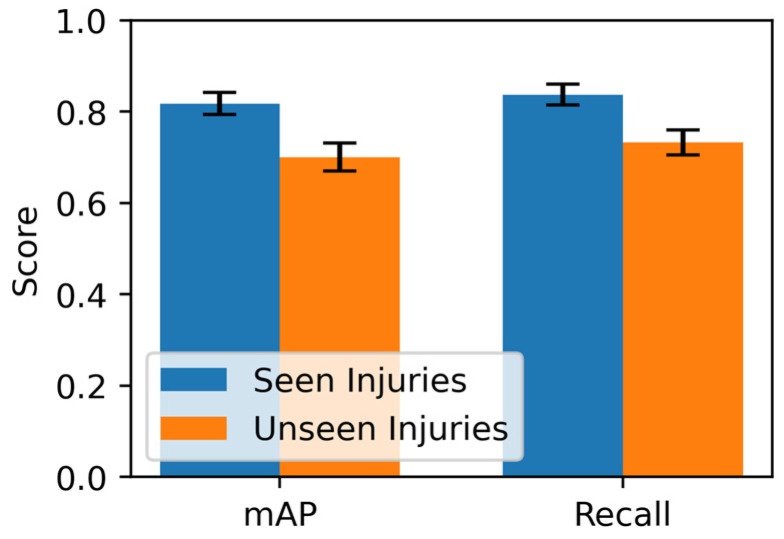
Model performance (mAP and Recall) for seen and unseen injuries at 0.5 IoU and 0.5 confidence. Error bars represent 95% confidence intervals obtained via bootstrapping.

To further assess partitioning effects, model performance was compared on a balanced test set of seen injuries (n = 968) versus a held-out set of unseen injuries (n = 968), filtered for matching acquisition conditions (Figure 13). At 0.5 IoU and 0.5 confidence, mAP declined from 0.82 (95% CI: [0.79, 0.84]) on seen injuries to 0.70 (95% CI: [0.67, 0.73]) on unseen injuries (a 15% relative drop), with recall decreasing from 0.84 to 0.73. Threshold sweeps confirmed consistent gaps, with mAP on unseen data dropping below 0.10 at strict thresholds (>0.7 confidence), mirroring similarity analysis trends.

These findings demonstrate that image-level partitioning, particularly under controlled acquisition conditions, can yield inflated generalization estimates. The performance degradation observed for less similar and unseen injuries highlights a degree of model overfitting to intra-injury visual patterns. While implementing strict injury-level partitioning is essential for rigorous benchmarking, a distinction must be made for real-world deployment. In operational scenarios, extrinsic factors (e.g., lighting, camera angle) introduce significant intra-class variance, meaning that different images of the same injury can present as novel challenges. Therefore, a model’s robustness to these environmental variations is a critical capability, distinct from its ability to generalize to physiologically novel injuries.

## 5. Limitations and Future Works

This study has several limitations. First, the experimental design was constrained by the available dataset, which featured a controlled imaging environment and known imbalances in skin tone representation. While balancing was applied, more advanced data augmentation strategies were not explored. Second, the evaluation was restricted to a selection of established CNN-based architectures and did not include other prominent frameworks like Vision Transformers or the YOLO series. Finally, hyperparameter optimization was limited by computational resources, and the evaluation relied on standard object detection metrics (e.g., mAP) that may not fully capture clinical or forensic relevance. Additionally, the 224 × 224 input resolution represents a downsampling trade-off between computational efficiency and precision. However, additional experiments at 480 × 480, 640 × 640, and 800 × 800 confirmed consistent model rankings and operational recommendations. Higher-resolution training is recommended for future deployment-oriented work.

Future work should proceed in three primary directions. (1) Data Curation and Partitioning: Priority should be given to developing more diverse datasets that encompass a wider range of patient demographics, injury etiologies, and severities under less controlled acquisition conditions. Crucially, future benchmarking must enforce strict injury-level partitioning to ensure an unbiased assessment of generalization to novel cases. (2) Methodological Expansion: The comparative analysis should be extended to include emerging architectures (e.g., Vision Transformers, large-scale foundation models including Vision-Language Models [60] and knowledge-distillation-based [61] approaches), as well as advanced augmentation techniques to mitigate dataset biases, alongside privacy-preserving approaches such as federated learning [62]. (3) Evaluation Frameworks: Future studies should develop and incorporate domain-specific evaluation metrics that are more closely aligned with forensic and clinical practice, moving beyond pixel-based measures to assess contextual relevance.

## 6. Conclusions

This work presents an operational evaluation framework for automated bruise detection that characterizes dataset- and model-dependent trade-offs relevant to forensic practice. Within the constraints of our dataset and controlled acquisition protocols, FCOS consistently delivered superior average precision under ALS-dominant training and exhibited more stable confidence distributions. Skin-tone balancing, while effective at lowering failure rates for darker skin tones, introduced increased overprediction in specific subgroups, underscoring a trade-off between sensitivity (detection coverage) and specificity (false-positive avoidance). Crucially, embedding-similarity and seen/unseen injury evaluations revealed substantive declines in detection performance on unseen injuries, indicating that image-level partitions can overstate generalization.

We therefore recommend three practical steps for researchers and practitioners: (1) prioritize ALS imaging during data collection when feasible; (2) adopt injury-level partitioning (or embed-similarity stratification) as a standard validation protocol to avoid optimistic generalization claims; and (3) perform model-specific operational tuning of confidence and IoU thresholds prior to deployment. Future work should extend the framework to multi-site datasets, explore localization-oriented loss/regularization to mitigate overprediction, and evaluate transformer-based detectors under the same protocol.

## Figures and Tables

**Table 1:** Distribution of full-consensus dataset across lighting conditions and filter settings.

Alias	Description	Total Samples	Train Samples	Validation/Test Samples
W	Light: White Filter: None	1046	732	157
ALS	Light: 415 nm & 450 nm Filter: all (O Y)	5406	3784	811
ALS+W	-	6452	4516	968

**Table 2:** Mean and standard deviation of RGB pixel values across datasets.

Dataset Name	Metric	R	G	B
W	Mean	0.505	0.356	0.300
	Std	0.315	0.230	0.199
ALS (415 nm & 450 nm)	Mean	0.393	0.314	0.017
	Std	0.256	0.273	0.021
-- with Orange Filter	Mean	0.453	0.227	0.006
	Std	0.294	0.185	0.004
-- with Yellow Filter	Mean	0.332	0.404	0.029
	Std	0.191	0.317	0.025
Combined (W + ALS)	Mean	0.411	0.321	0.063
(415 nm & 450 nm)	Std	0.269	0.267	0.133

**Table 3:** Data augmentation parameters.

Operation	Parameters
Resize	(224, 224)
Flip	H: *p* = 0.5 V: *p* = 0.5
Affine	Random crop: *p* = 0.5, ratio = 0.9 Rotate: *p* = 0.5
noise	Gaussian: *p* = 0.5, mean = 10, std = 50
normalize	mean = [0.485, 0.456, 0.406] std = [0.229, 0.224, 0.225]

**Table 4:** Train–test configurations for cross-dataset generalization evaluation. ☑ indicates tested configurations.

Train on Right → Test on Bottom ↓	W	ALS	ALS+W
W	☑		
ALS		☑	
ALS+W	☑	☑	☑

**Table 5:** AP of models across different train–test configurations (multi-threshold AP shown with mAP@0.5 in parentheses).

(Train Set) → (Test Set)			
	Faster R-CNN	FCOS	RetinaNet
(W) → (W)	0.332 (0.295)	0.392 (0.673)	0.335 (0.619)
(ALS) → (ALS)	0.152 (0.340)	0.584 (0.931)	0.306 (0.715)
(ALS+W) → (ALS+W)	0.174 (0.342)	0.531 (0.924)	0.310 (0.691)
(ALS+W) → (W)	0.200 (0.343)	0.443 (0.904)	0.320 (0.710)
(ALS+W) → (ALS)	0.180 (0.296)	0.541 (0.805)	0.315 (0.593)

**Table 6:** Model performance across different ALS/W ratios using FCOS.

(W Ratio)/(ALS Ratio) %	Avg of Values Across All IoUs and Confidence Threshold					
	AP (mAP@0.5)	CUM TP	CUM FP	Precision	Recall	F1 Score
0/100	0.4965 (0.849)	68	1804	0.3250	0.4402	0.3739
25/75	0.4724 (0.816)	69	2514	0.3004	0.4443	0.3585
50/50	0.4573 (0.774)	67	2088	0.2890	0.4307	0.3459
75/25	0.4065 (0.706)	60	2284	0.3060	0.3846	0.3408
100/0	0.3645 (0.686)	58	2582	0.2923	0.3769	0.3293

**Table 7:** Detection performance metrics for FCOS at different confidence and IoU thresholds (W/ALS = 0/100). NA indicates that no detections were made at the specified confidence threshold.

Confidence Thresh	IoU Thresh	AP	CUM TP	CUM FP	Precision	Recall	F1 Score
0.1	0.1	0.9563	156	8392	0.0182	1	0.0358
0.1	0.3	0.9491	156	8392	0.0182	1	0.0358
0.1	0.5	0.8486	156	8392	0.0182	1	0.0358
0.1	0.7	0.5204	111	8437	0.0129	0.7115	0.0255
0.1	0.9	0.0022	4	8544	0.0004	0.0256	0.0009
0.3	0.1	0.9552	155	491	0.2399	0.9935	0.3865
0.3	0.3	0.9480	155	491	0.2399	0.9935	0.3865
0.3	0.5	0.8429	149	497	0.2306	0.9551	0.3715
0.3	0.7	0.5187	108	538	0.1671	0.6923	0.2693
0.3	0.9	0.0022	4	642	0.0061	0.0256	0.0099
0.5	0.1	0.8652	137	19	0.8782	0.8782	0.8782
0.5	0.3	0.8592	136	20	0.8717	0.8717	0.8717
0.5	0.5	0.7483	125	31	0.8012	0.8012	0.8012
0.5	0.7	0.4811	93	63	0.5961	0.5961	0.5961
0.5	0.9	0.0022	4	152	0.0256	0.0256	0.0256
0.7	0.1	0.1089	17	0	1	0.1089	0.1965
0.7	0.3	0.1089	17	0	1	0.1089	0.1965
0.7	0.5	0.1089	17	0	1	0.1089	0.1965
0.7	0.7	0.1025	16	1	0.9411	0.1025	0.1849
0.7	0.9	0.0007	1	16	0.0588	0.0064	0.0115
0.9	0.1	NA					
0.9	0.3	NA					
0.9	0.5	NA					
0.9	0.7	NA					
0.9	0.9	NA					

**Table 8:** Effect of balancing dataset on prediction performance across skin colors. U: Unbalanced, LFb: Light/Filter-balanced, Sb: Skin-balanced.

Skin Color	Dataset Config	Prediction Status (%)			
		Failed	Overpredict	Perfect	Underpredict
Very Light	U	8.48	2.42	87.27	1.82
	Sb	3.31	10.74	82.64	3.31
	LFb	4.41	10.29	77.94	7.35
Light	U	7.00	4.00	82.00	7.00
	Sb	6.87	12.98	70.23	9.92
	LFb	7.41	10.19	70.37	12.04
Intermediate	U	8.51	3.19	84.04	4.26
	Sb	1.67	5.83	83.33	9.17
	LFb	6.76	10.81	70.27	12.16
Tan	U	14.99	6.24	73.75	5.00
	Sb	7.69	17.69	68.46	6.15
	LFb	12.09	4.40	76.92	6.59
Brown	U	12.31	3.85	76.92	6.93
	Sb	2.92	18.25	74.45	4.38
	LFb	4.62	16.92	69.23	9.23
Dark	U	14.87	2.48	72.72	9.92
	Sb	5.56	11.90	78.57	3.97
	LFb	13.56	10.17	64.41	11.86

**Table 9:** Statistical comparison of models across skin-tone subgroups for Failed cases. 95% Wilson score confidence intervals; Fisher’s exact test (two-sided per subgroup). Significant threshold = 0.05.

Skin Color	U (n)	U Fail % (95% CI)	Sb (n)	Sb Fail % (95% CI)	*p*-Value	Significant
Very Light	165	8.5% (5.1–13.7%)	121	3.3% (1.3–8.2%)	0.088	no
Light	200	7.0% (4.2–11.4%)	131	6.9% (3.7–12.5%)	1.000	no
Intermediate	188	8.5% (5.3–13.4%)	120	1.7% (0.5–5.9%)	0.012	yes
Tan	160	15.0% (10.3–21.4%)	130	7.7% (4.2–13.6%)	0.066	no
Brown	130	12.3% (7.7–19.1%)	137	2.9% (1.1–7.3%)	0.004	yes
Dark	121	14.9% (9.6–22.3%)	126	5.6% (2.7–11.0%)	0.019	yes

**Table 10:** Statistical comparison of models across skin-tone subgroups for Overprediction cases. 95% Wilson score confidence intervals; Fisher’s exact test (two-sided per subgroup). Significant threshold = 0.05.

Skin Color	U (n)	U Overp. % (95% CI)	Sb (n)	Sb Overp. % (95% CI)	*p*-Value	Significant
Very Light	165	2.4% (0.9–6.1%)	121	10.7% (6.4–17.5%)	0.004	yes
Light	200	4.0% (2.0–7.7%)	131	13.0% (8.3–19.8%)	0.005	yes
Intermediate	188	3.2% (1.5–6.8%)	120	5.8% (2.9–11.5%)	0.384	no
Tan	160	6.2% (3.4–11.1%)	130	17.7% (12.1–25.2%)	0.003	yes
Brown	130	3.8% (1.6–8.7%)	137	18.2% (12.7–25.5%)	<0.001	yes
Dark	121	2.5% (0.8–7.0%)	126	11.9% (7.4–18.7%)	0.006	yes

**Table 11:** Detection outcome percentages across different lighting and filter conditions.

	W (%)	ALS (%)			
	No filter	450 nm O	450 nm Y	415 nm O	415 nm Y
Invalid Failed Consistent	15.29	5.44	4.97	5.18	6.01
Invalid Failed Inconsistent	10.59	0	1.99	1.88	4.37
Valid Overpredict Consistent	1.18	0.49	0.49	1.41	2.18
Valid Overpredict Inconsistent	6.47	0.49	3.48	1.41	1.63
Valid Perfect Consistent	30.59	74.75	68.15	70.75	65.57
Valid Perfect Inconsistent	25.29	13.36	14.92	15.56	16.93
Valid Underpredict Consistent	4.71	3.96	4.47	2.83	2.73
Valid Underpredict Inconsistent	5.88	1.48	1.49	0.94	0.54

**Table 12:** Detection performance percentages (%) across different skin tones.

	Very Light	Light	Intermediate	Tan	Brown	Dark
Invalid Failed Consistent	7.27	5	6.38	8.12	8.46	8.26
Invalid Failed Inconsistent	1.21	2	2.13	6.87	3.85	6.61
Valid Overpredict Consistent	0	1	0.53	3.12	0.77	1.65
Valid Overpredict Inconsistent	2.42	3	2.66	3.12	3.08	0.83
Valid Perfect Consistent	64.85	59.5	69.15	56.25	66.92	61.98
Valid Perfect Inconsistent	22.42	22.5	14.89	17.5	10	10.74
Valid Underpredict Consistent	1.21	4.5	2.66	3.75	3.08	7.44
Valid Underpredict Inconsistent	0.61	2.5	1.6	1.25	3.85	2.48

**Table 13:** Classification accuracy and clustering performance of FCOS model trained on ALS+W.

Model	Classification			Clustering		
	Decision Tree	Random Forest	XGBoost	Sil. Score	ARI	CH Index
FCOS	0.66	0.82	0.78	0.27	0.010	393.95

## Data Availability

Some or all data, models, or codes that support the findings of this research are available from the corresponding author upon reasonable request.
